# Reversing Mitochondrial, Metabolic and Molecular Defects in the Brain Improves Cognition in Aged Mice

**DOI:** 10.1093/geroni/igaa057.3158

**Published:** 2020-12-16

**Authors:** Rajagopal Sekhar, Premranjan Kumar

**Affiliations:** Baylor College of Medicine, Houston, Texas, United States

## Abstract

Age-associated cognitive-decline is a risk factor for Alzheimer’s disease (AD), but mechanisms are not well understood, and interventions are lacking. Rodent studies on AD have not led to therapeutic breakthroughs for cognitively-impaired humans. In an open-label trial in older-adults we found that supplementing GlyNAC (glutathione precursors glycine and N-acetylcysteine) improved cognitive-decline, defects in whole-body mitochondrial-function, and systemic insulin-resistance, oxidative-stress, and inflammation. We hypothesized that aged-mice will have similar defects in the brain, and studied male C57BL/6J mice as follows: young-mice (20w) were compared to two-groups of aged-mice (90-weeks) receiving either GlyNAC or isonitrogenous-placebo diets for 8-weeks. GlyNAC-supplementation improved cognition, and the following measures in the brain: glutathione-concentrations, glucose-transporters in blood-brain-barrier and neurons, mitochondrial glucose-oxidation, oxidative-stress, endoplasmic-reticulum stress, autophagy, mitophagy, inflammation, senescence, genomic and telomere damage. These data provide mechanistic insights into the novel and beneficial role of GlyNAC supplementation to reverse cognitive-decline in aging, and holds promise for human AD.

